# Adherence to and appropriateness of anti-osteoporotic treatments in patients aged 50 and over in the Valencia Region (Spain). The ESOSVAL-AD Study

**DOI:** 10.1186/1471-2474-12-178

**Published:** 2011-08-03

**Authors:** Gabriel Sanfélix-Gimeno, José Sanfélix-Genovés, Salvador Peiró, Isabel Hurtado, José Luis Trillo, Ruth Usó, Vicente Giner Ruiz, Manuel Pascual de la Torre, Inmaculada Ferreros

**Affiliations:** 1 Centro Superior de Investigación en Salud Pública (Center for Public Health Research).Valencia, Spain; 2 Centro de Salud de Nazaret, Departamento València-Clínic-Malvarrosa, Agencia Valenciana de Salud. Valencia, Spain; 3 Dirección General de Farmacia y Productos Sanitarios, Agencia Valenciana de Salud, Valencia, Spain; 4 Centro de Salud Ciudad Jardín, Departamento de Alacant-Hospital General, Agencia Valenciana de la Salud. Alicante, Spain; 5 Oficina de Abucasis, Agencia Valenciana de Salud, Valencia, Spain

## Abstract

**Summary:**

**Background**

A study to evaluate the adherence to and appropriateness of anti-osteoporotic treatments in a cohort of men and women aged 50 and over participating in the ESOSVAL-R study.

**Methods/Design**

*Design*: An observational, longitudinal, prospective cohort study; Study subjects: Men and women aged 50 and over living in the Valencia Region (Spain) who initiated treatment between June 15, 2009, and June 15, 2011, in primary healthcare centers with electronic medical records; *Data sources*: The main data source will be electronic medical records. *Measurement of results: *Degree of compliance with and persistence of anti-osteoporotic treatments, and the proportion of patients with appropriate anti-osteoporotic treatment in accordance with the most relevant and high impact recommendations with clearly defined treatment algorithms in Spain (the Spanish National Health System guide (2010), the General Practitioners' Society (2007) and the General Directorate for Pharmacy and Medical Products of Madrid (2007)), and with the National Osteoporosis Foundation (NOF, 2010), and the International Osteoporosis Foundation guidelines (IOF, 2008); *Analysis*: 1.) Descriptive analysis of patients undergoing treatment and the treatments prescribed; 2.) Descriptive analysis of compliance with and persistence of anti-osteoporotic treatments; 3.) Analysis of factors associated with compliance with and persistence of treatments by Cox proportional hazard regression models, 4.) Descriptive analysis of appropriateness of treatment; 5.) Analysis of factors associated with the appropriateness of treatment by multilevel models (4 levels: patient, doctor, Basic Healthcare Zone/Primary Healthcare Center, and Health Area variables).

**Discussion**

ESOSVAL-AD will provide information regarding adherence to osteoporosis treatments and the factors associated with a higher or lower adherence (including the appropriateness of the treatment) in the Spanish context. A better understanding of this phenomenon and the interventions needed to address it would contribute to the increased effectiveness of therapeutic measures, a reduction in morbidity and mortality, and a corresponding reduction in healthcare costs.

## Background

Osteoporotic fractures constitute a serious health problem not only because of their severe consequences for patients in terms of pain and limited function, but also because of their important social and economic repercussions. Although many issues remain to be clarified [1], numerous studies have associated osteoporotic fractures (hip and also vertebral fractures) with higher mortality rates [2-4].

Osteoporosis is thus a high priority health problem. Paradoxically, although effective, well-tolerated treatments for managing this condition through fracture risk reduction are available in Spain, the Clinical Practice Guidelines (CPGs) of the various scientific societies that focus on this health problem (SEMFYC, SEMI, SER, SEMERGEN, SEIOMM, SECOT) give no recommendations for deciding when to initiate treatment based on fracture risk probability estimates. The proposal made by Vázquez et al. (2007) [5] is the only one that establishes treatment thresholds based on 10-year fracture risk estimates, both for vertebral and hip fractures.

Internationally, the most relevant and influential risk scale is that developed by the World Health Organization (FRAX). This scale estimates the risk of major osteoporotic fractures (vertebrae, hip, wrist, or humerus) as well as of hip fractures alone. Based on this scale, Kanis [6] and Dawson-Hughes [7] (IOF/NOF) have proposed intervention thresholds for the UK and the US for establishing treatment for major osteoporotic fractures as well as vertebral fractures and these have been included in the NOF recommendations. Recently, Hippisley-Cox (2009) [8] proposed an algorithm validated in the English population for estimating the individual 10-year risk for osteoporotic fractures (vertebrae, hip, or wrist) and hip fractures which predicts fracture risk without the need for complementary examinations.

The current situation, which combines scarce estimations of fracture risk with uncertainty and controversy concerning treatment candidates, has led to wide variability in the therapeutic management of osteoporosis. This variability encompasses undertreatment in high fracture risk patients as well as overtreatment in patients with a low fracture risk. With regard to the latter, a recent study carried out in a primary healthcare center in Madrid [9] found that 48% of patients treated with antiresorptive drugs did not meet the criteria for receiving treatment as set out in the CPGs most frequently used in Spain (developed by the scientific societies of various specialties: SER, SEIOMM, SEMFyC, SEMI, SECOT, SEMERGEN).

Along with this problem of the inappropriateness of treatment (including both under and overuse of certain drugs), treatments effectiveness for chronic diseases has the added difficulty of a patient's adherence to that treatment. Because response to treatment is related to both the dose and the administration regimen of drugs, non-compliance with treatment may reduce its benefits [10] and may also reduce its effectiveness [11,12]. Nevertheless, a low level of compliance with medical prescriptions is quite prevalent. Although a global quantification of non-compliance is difficult due to its variations depending on the disease and also with regard to the different therapeutic indications, it is estimated to reach 50% for therapeutic indications in chronic diseases [13].

Few studies have analyzed the adherence to treatments involving antiresorptive drugs. A recent systematic review of the literature [14] found an adherence rate of 67% for the first year of treatment, with an average persistence of 180 days of treatment/year (the review did not include any studies carried out in Spain). The few Spanish studies that have been published to date show similar or slightly lower compliance rates; however, they are difficult to assess as they included interventions to improve compliance. With respect to associated treatments, one recent study carried out in Spain estimated that compliance with taking calcium and/or vitamin D supplements was around 50% [15]. In contrast, a study conducted in our setting with regard to Hormone Replacement Therapy (prior to the publication of the WHI clinical trial), estimated that there was a 75% probability that the women in the study would comply with the therapy for 5 years [16].

The lack of studies in this field contrasts with the high relevance of knowing the rate of adherence to osteoporosis treatments and the factors associated with a higher or lower adherence (including the appropriateness of the treatment) in each specific context. This is indeed a relevant research aim given that a better understanding of this phenomenon and the interventions needed to address it could contribute to the increased effectiveness of therapeutic measures, a reduction in morbidity and mortality, and a corresponding reduction in healthcare costs [17].

In our setting an observational, prospective cohort study (the ESOSVAL-R study) is currently being carried out, in which 14,500 men and women will be followed-up for 5-10 years with the collaboration of 800 health care professionals from the Valencia Health Agency. Its objective is to develop a predictive risk scale for osteoporotic fractures for the adult population of the Valencia Region, to evaluate its validity, and to generate the information necessary for defining treatment criteria based on the fracture risk [18].

Within this cohort, which will be followed-up primarily with the aid of data from the electronic medical records system (ABUCASIS), which has just been redesigned, as well as from the electronic prescription system (GAIA), it will be possible to nest a second prospective cohort made up of patients who have recently begun treatment for osteoporosis. This cohort will be followed up to evaluate the adherence to and appropriateness of treatments as well as to analyze the factors associated with adherence. The aim of the present research project (ESOSVAL ADherence/Appropriateness or ESOSVAL-AD) is to evaluate the adherence (compliance and persistence) to and the appropriateness of anti-osteoporotic treatments in a cohort (nested within the ESOSVAL-R cohort) of patients who initiated these treatments within 2009 and 2010.

## Methods/Design

### Main Objective

To describe the adherence to and appropriateness of the indications of anti-osteoporotic treatments in a cohort of men and women aged 50 and over who began an anti-osteoporotic treatment in 2009-2010 and who were included in the ESOSVAL-R cohort; and to analyze the relationships between adherence and appropriateness as well as between fracture risk and adherence and appropriateness.

### Specific objectives

1. To describe the sociodemographic and clinical characteristics of the cohort.

2. To describe the treatments administered stratifying by age, sex, and certain clinical characteristics (presence of vertebral fracture, comorbidity, polypharmacy, fracture risk).

3. To evaluate the adherence (compliance and persistence) to anti-osteoporotic treatment in global terms as well as specifically for appropriate and inappropriate indications.

4. To evaluate the appropriateness of the anti-osteoporotic treatments.

5. To analyze the factors (patient, professional, organizational) associated with treatment appropriateness using multilevel models.

6. To analyze the factors associated with persistence and compliance with anti-osteoporotic therapies using proportional risk regression models.

### Design

This is an observational, longitudinal, prospective cohort study with a minimum follow-up period of two years. The data used in the study will be obtained primarily from the electronic clinical records system (ABUCASIS), specifically from the electronic prescription system (GAIA) and the ESOSVAL module which was designed for the management and follow-up of osteoporotic patients. To evaluate persistence with anti-osteoporotic treatment, patients will be followed up from the time of their inclusion in the study until June 15, 2013.

### Setting

The sample will be recruited from the Valencia Region, Spain (Primary Care practices of the Valencia Health Agency whose professionals are participating in the ESOSVAL-R project. This includes 450 practices and 800 healthcare professionals.)

### Population and sample

The characteristics of the ESOSVAL cohort have been amply described elsewhere [18]. In brief, the cohort comprises 14,500 men and women aged 50 and over who live in the Valencia Region and who were recruited opportunistically from among the patients who attended the collaborating primary care centers. Excluded from the study were those patients with cognitive impairments, those not insured through the Valencia Health Agency (e.g. members of civil servant insurance mutuals), people who are physically unable to attend their usual primary healthcare center, non-residents of the Valencia Region, and people of Asian or African descent. From the selected study population, a smaller nested cohort (ESOSVAL-AD) was chosen to include patients who had initiated anti-osteoporotic treatment in the 12 months prior to or after their inclusion in the general cohort (between June 15, 2009, and June 15, 2011). In order to make the study population more homogeneous by avoiding the inclusion (as initial cases) of treated patients who had discontinued treatment during any given month, patients who had received anti-osteoporotic treatment in the 6 months prior to being selected were excluded from the ESOSVAL-AD cohort.

Estimates indicated the possibility of obtaining an *n *of approximately 500 subjects who had initiated treatment in the ESOSVAL-AD inclusion period, which is somewhat higher than the 330 required to estimate the main endpoint of the study with a precision of ± 5 (alpha: 0.05, power: 0.80), but useful for improving the power of the secondary analyses.

### Outcomes

#### Main endpoint

Extent of compliance with the anti-osteoporotic treatment prescribed, calculated as the number of pills dispensed in relation to the number of pills necessary for treatment during the time period evaluated. Good compliance is when a patient has picked up more than 80% of the prescribed drug from the pharmacy during the corresponding follow-up period.

#### Secondary endpoints

1) Persistence of medication is defined as the time elapsed between the start of a given treatment and its interruption [19]. With regard to this variable, treatment starts when a patient receives a prescription for a given anti-osteoporotic drug, always when the patient has not received any other prescriptions for anti-osteoporotic drugs in the previous 6 months. Treatment ends when the patient has not received the prescribed anti-osteoporotic drug for three months (90 days time lag). 2) Proportion of patients with appropriate anti-osteoporotic treatment. Treatment appropriateness was assessed according to the most relevant and high impact recommendations with clearly defined treatment algorithms in Spain (the Spanish National Health System guide (2010) [20], the General Practitioners' Society (2007) [21] and the General Directorate for Pharmacy and Medical Products of Madrid (2007) [22]), and to the recommendations of the National Osteoporosis Foundation (NOF, 2010) [23], and the International Osteoporosis Foundation guidelines (IOF, 2008) [24].

### Others variables and definitions

▪ *Of the patients: *age, sex, height and weight with a body mass index calculation, smoking habits, consumption of alcohol, exercise habits, antecedents of first degree family member with hip fracture, low calcium intake, non-treated hypogonadism, rheumatoid arthritis, other diseases that decrease bone mass (excluding hypogonadism), use of oral glucocorticoids, drugs that decrease bone mass (excluding glucocorticoids), previous osteoporotic fracture, high risk of fall, prolonged immobilization, osteoporosis of the lumbar spine assessed by DXA (T score for L2-L4), osteoporosis of the hip assessed by DXA for the whole hip or femur neck (T score), concomitant medication and morbidity, and mortality.

▪ *Of the primary care doctor*: age, sex, training (whether she/he gained their Family Physician certificate after a nationally accredited 3-4 year residency program), work situation (fixed employment/other undefined issues), time working in primary care (years), time in current position (years), specialty.

▪ *Of the Institution: *Primary Healthcare Center, Basic Healthcare Zone, and Health Department.

### Data sources

The main source of data will be the ABUCASIS electronic clinical records which, apart from clinical and sociodemographic data about the patient, contain all the information pertaining to the prescription and dispensation of drugs (electronic prescription system-GAIA) in ambulatory care (primary and specialized care). To identify those patients who either die or move to other Regions of Spain (censured cases); this data source will be complemented by the Population Information System of the Valencia Health Ministry.

Baseline data will be collected during the initial visit, using a new modified version of the ABUCASIS specifically developed to collect information about the variables used in the study that were not routinely found in clinical records. The results of previous examinations (x-rays or densitometry) that may have been performed on the patients will be included in the initial evaluation (information about previous fractures and densitometry values). In the case of densitometry results, the information that will be included in the initial evaluation will be from examinations done in a period of ± 2 years at the time of recruitment.

### Statistical Analysis

1) Once the recruiting process has been completed, the cohort baseline data will be analyzed. This will include the description of the characteristics of the study subjects, the treatments employed, and treatment appropriateness. The appropriate parameters (means, proportions) will be used with each variable with their corresponding 95% confidence intervals (CI95%).

2) At the end of the follow-up period, a descriptive analysis of compliance with and persistence of anti-osteoporotic treatments will be carried out. This analysis will also be stratified according to the degree of appropriateness and FRAX scales.

3) Next, Cox proportional hazards models will be used to evaluate the independent factors associated with compliance with and persistence of treatment.

4) Finally, a hierarchical analysis (multilevel) will be carried out with respect to the appropriateness variable on 4 levels: 1.) Clinical and demographic variables of the patient, 2.) Doctor variables, 3.) Variables of the Basic Healthcare Zone/Primary Healthcare Center and 4.) Health Area.

The analyses will be carried out with the aid of the STATA v. 10 and R statistical packages.

### Ethical aspects

#### Interventions derived from the study

This observational study will be carried out under the usual conditions of clinical practice and in accordance with best clinical practices. No test or treatment derived solely from this study (over and above a detailed medical history which will be collected as part of the usual electronic medical records) will be carried out.

#### Compliance with the standards of good research practices

This study will be conducted in accordance with the international standards for epidemiological studies, as established in the International Guidelines for Ethical Review of Epidemiological Studies (Council for the International Organizations of Medical Sciences-CIOMS-Geneva, 2009) and with the recommendations of the Spanish Epidemiological Society on the review of ethical issues in epidemiological research.

#### Committee for Ethics and Clinical Trials

This study shares all the aspects of data sources, data management, and confidentiality with the ESOSVAL-R study, which has already been approved by the Committee for Ethics and Clinical Trials of the Center for Public Health Research and the Public Health Administration (CEIC CSISP-DGSP). In addition, all the study subjects have given their informed consent for access to anonymized data in the databases containing their medical records. Likewise, the participating centers have signed the corresponding informed agreements concerning their collaboration in the study. Nevertheless, in accordance with the Resolution of the Regional Health Secretary dated December 15, 2009, with regard to the request for data, treatment of data, and release of data from the ABUCASIS system (SIA-GAIA), a request for any new variable in the ABUCASIS system requires separate and renewed approval by the CEIC. This protocol has also been approved by the CEIC CSISP-DGSP.

## Discussion

Like all observational studies, ESOSVAL-AD has several limitations which we have tried to minimize. Those most noteworthy include:

### 1. Selection bias

Although a certain degree of selection bias is inevitable (the elderly and the very ill cannot physically get to the Primary Healthcare Center as often as others), it is also possible that collaborating researchers select study subjects who are easier to interview (e.g. with a higher cultural level) and avoid choosing other candidates. To reduce this bias, we will highlight its importance in our training sessions and we have also prepared a selection scheme based on surgery timetabling and the scheduling of the patients.

### 2. Information bias due to the absence of registry or differential of registry of data in the electronic clinical records

Although this problem is ever present when a study is based on data from real clinical practices, various strategies will be applied to minimize its effect: a.) To guarantee that all the participating clinicians are up to date on the topic of osteoporosis, that during medical visits they all respond in a similar fashion and that the quality of the electronic clinical records is as high as possible, all the participating clinicians (800) will take part in a 300-hour training course, both in-class and on line, on the management of osteoporosis patients. The course is to be offered through the Valencia School of Health Studies and accredited by the National Health Service; b.) The electronic clinical records system has also been modified to improve data collection and unify the registry with regard to initial visits as well as during follow up. These improvements in the electronic medical records system apply to all healthcare professionals in the health system, and not only to those participating in this study.

### 3. Intervention and maturation bias

Due to the training sessions associated with the ESOSVAL project as well as to the fact that in an open study clinicians undergo a learning process, patients included in this study may be better monitored and receive more treatment than the general population. This could lead to differences not only in the prevalence of anti-osteoporotic treatments, but also in treatment appropriateness and adherence between cohort participants and the population as a whole. This possible bias is present in all open studies and is difficult to handle. In our case, given that the educational input is concentrated at the beginning of the study, we are hopeful that the impact of this bias will be minimized.

### 4. Measuring compliance

The assessment of compliance has been carried out with the aid of the electronic prescription system (GAIA) of the Valencia Health Ministry. Although this is a reliable system for evaluating compliance in terms of counting pills (number of pills dispensed/number of pills necessary for treatment in the evaluation period), it is not possible to know for certain whether the patient has actually taken the medicine in the prescribed dose and manner and is following the doctor's recommendations.

### 5. Anti-osteoporotic treatments

the information regarding medicines dispensed will be taken from the electronic prescription system (GAIA) included in ABUCASIS. This system only contains information from ambulatory care; therefore we don't have information about medicines dispensed in hospitals. The only drug affected by this limitation is zoledronic acid, and therefore we will not have information about it in this study.

## Abbreviations

ESOSVAL-R: Esosval Risk study; CPG: Clinical Practice Guidelines; NOF: National Osteoporosis Foundation; IOF: International Osteoporosis Foundation; DXA: dual energy x-ray absorptiometry; CIOMS: Council for the International Organizations of Medical Sciences; CSISP, Centro Superior de Investigación en Salud Pública; CEIC: Committee for Ethics; DGSP: Dirección General de Salud Pública;

## Competing interests

The authors declare that they have no competing interests.

## Authors' contributions

GSG, JSG and SP designed the study. JSG, IH, MPT, JLTM, RU, IF and VG contributed in several parts of the ESOSVAL-AD study (ABUCASIS modifications, database designs). All authors contributed to the writing of the manuscript, corrected draft versions and approved the final manuscript.

## Pre-publication history

The pre-publication history for this paper can be accessed here:

http://www.biomedcentral.com/1471-2474/12/178/prepub
